# Vitamin D status and overall and sepsis mortality in critically ill patients

**DOI:** 10.1186/cc12385

**Published:** 2013-03-19

**Authors:** P Zajic, C Schnedl, T Valentin, A Grisold, A Holl, S Farzi, S Fruhwald, H Dobnig, TR Pieber, K Amrein

**Affiliations:** 1 Medical University of Graz, Austria

## Introduction

Vitamin D status may affect hospital mortality and infectious complications in critical illness.

## Methods

A total of 655 mixed critically ill patients with available 25(OH)D levels hospitalized between 2008 and 2010 were included. Cox regression analysis adjusted for SAPS 2, age and gender was performed. 25(OH)D levels were categorized by3month-specific tertiles (high, intermediate, low) to reflect seasonal variation. Primary endpoint was the correlation between 25(OH)D and hospital mortality. Secondary endpoints were sepsis mortality and blood culture positivity.

## Results

Hospital mortality was significantly higher in patients in the low (HR = 1.98) and the mid-range tertile (HR = 1.88) compared with the highest tertile. Vitamin D levels were significantly lower in patients with lethal sepsis compared with other causes of death (12.3 ± 5.0 vs. 18.2 ± 11.2, *P *= 0.02). Blood culture positivity rates did not differ between the groups (23.0% vs. 26.8% vs. 17.3%, *P *= 0.361). See Table 1.

**Table 1:** 

	Low	Intermediate	High	
Tertile	(*n *= 216)	(*n *= 219)	(*n *= 220)	*P *value
25(OH)D (ng/ml)	10.4 (± 3.6)	17.6 (± 5.1)	30.7 (± 11.4)	<0.001
SAPS 2	32 (± 15)	28 (± 16)	28 (± 16)	0.007
Hospital mortality	58 (26.9%)	48 (21.2%)	29 (13.2%)	0.002
Lethal sepsis	11 (5.1%)	9 (4.1%)	0 (0.0%)	0.005

## Conclusion

Low 25(OH)D status is predictive of all-cause and sepsis mortality in the critically ill. Interventional studies are needed to investigate the effect of vitamin D on mortality and sepsis incidence and outcomes.

